# Anti-inflammatory and anti-oxidant action of tadalafil in testicular regeneration process after heat stress

**DOI:** 10.1590/1984-3143-AR2023-0095

**Published:** 2024-06-21

**Authors:** Fernando Leonel da Silva, Fernanda Carolina Ribeiro Dias, Sandra Maria Torres, Virginia Maria Barros de Lorena, Sebastião Rogerio de Freitas Silva, Vinicius Vasconcelos Gomes de Oliveira, Emanoel Felipe de Oliveira, Pierre Castro Soares, Valdemiro Amaro da Silva

**Affiliations:** 1 Departamento de Medicina Veterinária, Universidade Federal Rural de Pernambuco, Recife, PE, Brasil; 2 Instituto Superior de Estudos Interculturais e Transdisciplinares de Viseu, Instituto Piaget, Viseu, Portugal; 3 Departamento de Imunologia, Instituto Aggeu Magalhães, Fundação Oswaldo Cruz, Recife, PE, Brasil; 4 Departamento de Nutrição, Universidade Federal de Pernambuco, Recife, PE, Brasil

**Keywords:** heat stress, testis, tadalafil, wistar rats, phosphodiesterase

## Abstract

Tadalafil, a potent phosphodiesterase inhibitor 5 (PDE-5), is commonly used for the management of erectile dysfunction. However, its therapeutic potential extends beyond this indication. This study aimed to investigate the impact of tadalafil on the recovery of testicular parenchyma in male Wistar rats exposed to testicular thermal stress. Fifty-four Wistar rats were subjected to testicular thermal stress and randomly assigned to receive either tadalafil treatment (TAD) or no treatment (control). TAD was administered intraperitoneally at a dose of either 0.9 mg/kg or 1.8 mg/kg. Biometric parameters, histopathological assessment of the testis, serum testosterone levels, oxidative stress, and interleukin levels were evaluated on days 7, 15, and 30 after thermal shock. The animals were euthanized at the end of each experimental period, and samples were collected. TAD treatment maintained testicular weight and reduced the testicular degenerative process up to day 7 post-injury. However, despite TAD therapy, serum testosterone levels were decreased in the treated groups at days 7 and 15 post-thermal stress. TAD also decreased TNF-α and NO levels at different doses but had no effect on IL-6. The treatment with TAD after heat shock demonstrated anti-inflammatory and antioxidant properties but did not prevent the aggravation of testicular lesions in subsequent periods, even with the systematic reduction in TNF-α and NO levels. Therefore, this selective PDE-5 inhibitor, at the dosages used, did not have a positive impact on testosterone levels during the post-thermal stress period, which could compromise the resumption of the spermatogenic process.

## Introduction

The testes are essential organs for species perpetuation, located outside the abdominal cavity, and exposed to temperatures ranging from 2 ºC to 6 ºC below body temperature (Kastelic et al., 1997; Queiroz et al., 2013). The vascular cone, formed by the veins of the pampiniform plexus surrounding the testicular artery, regulates temperature through countercurrent heat exchange, blood flow regulation, and heat loss by irradiation (Queiroz et al., 2013). The scrotum’s thin skin, poor subcutaneous fat, well-developed blood and lymphatic systems, and numerous sweat glands further contribute to thermal regulation. Despite these physiological mechanisms, heat stress-induced male gonad degeneration can still occur (Kastelic et al., 1997; Lue et al., 1999).

Spermatogenesis suppression is a reversible effect of testicular warming in several mammalian species, including humans and rats (Rockett et al., 2001; Zhang et al., 2014; Fang et al., 2019). Testicular cells are particularly vulnerable to heat stress, and supra-scrotal temperatures induce cell death via apoptosis, mediated by the tumor gene suppressor protein p53, a potent biochemical mediator of heat stress-induced apoptosis (Kumagai et al., 2002; Aldahhan and Stanton, 2021). Additionally, germ cells increase heat shock protein (HSP) synthesis in response to heat stress (Yin et al., 1997; Conti, 2000; Aldahhan and Stanton, 2021).

PDE-5 inhibitors have been shown to reduce intracellular cytochrome C and modulate the Bcl-2/Bax9 ratio, thereby modulating the reduction of apoptotic cells in induced tissue injury. Testicular tissue reperfusion injuries promote an increase in the expression of iNOS, eNOS, and NO to toxic levels, resulting in apoptosis of testicular germ cells, but tadalafil administration reduces this increase (Üstün et al., 2008; Erol et al., 2009).

Direct application of heat to the testicles has revealed new insights into the triggering mechanisms of damage to spermatogenesis and potential treatments to prevent damage by blocking testis apoptosis, according to Setchell (Setchell, 2006). However, no prior studies have investigated the effects of PDE-5 inhibitors on spermatogenesis or sperm regeneration after heat shock. Given tadalafil’s demonstrated cell protection and apoptosis inhibition properties, this study analyzed the effects of this PDE-5 inhibitor on spermatogenesis recovery after testicular degeneration induced by heat shock.

## Material and methods

### Experimental draw

Ninety-day-old male Wistar rats (*Rattus norvegicus*, var. Albinos) obtained from the vivarium of the Department of Animal Morphology and Physiology of the Federal Rural University of Pernambuco were utilized for this study. The rats were housed in a controlled environment with standard conditions of temperature (22 ºC), humidity (50%), and a 12-hour light/dark cycle. Standard solid food (Labina Purina) and tap water were provided ad libitum.

The experimental protocol was approved by the Ethics Committee for the Use of Animals of the Federal University of Pernambuco (CEUA/UFPE), registered under nº 0028/2016, and the study was conducted at the Academic Center of Vitória (CAV-UFPE).

The rats were randomly assigned to one of the three experimental groups: thermal shock group (n=18); thermal shock group treated with a daily intraperitoneal dose of 0.9 mg/kg^-1^ of tadalafil (TAD) (n=18); thermal shock group treated with a daily intraperitoneal dose of 1.8 mg/kg^-1^ of TAD (n=18).

The animals were weighed daily to calculate the drug doses. The animals were initially anesthetized with ketamine (Vetanarcol, 100 mg/kg) and xylazine (Kensol, 5 mg/kg) by intraperitoneal injection, and their testicles and tail were then immersed for 15 minutes in water at a temperature of 43ºC according to the protocol established by Queiroz et al. (2013).

After thermal shock, the rats were transferred to their cages (n = 6) and kept at room temperature until they recovered from anesthesia. The following day, the rats began to receive daily intraperitoneal applications of distilled water (control), 0.9 or 1.8 mg/kg^-1^ of TAD, according to their respective experimental group. The body weight was measured daily to calculate the TAD doses. The experimental analyses were performed 7, 15, and 30 days after the heat shock.

### Testicular perfusion

At the conclusion of the experimental period, the rats were administered heparin (125 UI/100 g; Akzo Organon Teknika) and anesthetized with Thiopental (50 mg/Kg; Roche) before undergoing intracardiac perfusion with a 0.9% NaCl solution containing heparin (500 UI/L) and sodium nitroprusside (100mg/L; Sigma). Next, the rats were perfused with glutaraldehyde 4% (Vetec) in sodium phosphate buffer (pH 7.2 and 0.01M). Following fixation, the testes were extracted and weighed using a BEL Engineering balance (MARK 500 / BRA) with a precision of 0.001g.

### Testicular histopathology

At the conclusion of the experimental period, the rats were subjected to heparinization (125 UI/100 g; Akzo Organon Teknika), anesthesia (Thiopental 50 mg/Kg; Roche), and intracardiac perfusion with a solution containing 0.9% NaCl, heparin (500 UI/L), and sodium nitroprusside (100 mg/L; Sigma). Following this, glutaraldehyde 4% (Vetec) in sodium phosphate buffer (pH 7.2 and 0.01M) was used to perfuse all rats. The testes were then extracted and weighed using a BEL Engineering balance (MARK 500 / BRA) with a precision of 0.001 g.

Subsequently, testicular fragments of 2 mm thickness were sliced and re-fixed for an hour in the same perfusion solution. Afterward, they were soaked in phosphate buffer for two hours, dehydrated in a series of crescent alcohol concentrations, and embedded in a plastic resin consisting of glycol methacrylate (LEICA). Histological sections of 4 µm thickness were obtained and stained with 1% toluidine blue / sodium borate for morphological and morphometric analyses. The testicular components were evaluated histopathologically, as per the protocol established by Queiroz et al. (2013), using an optical microscope (Leica DM500E).

### Determination of lipid peroxide levels in liver and spleen homogenate

At the conclusion of the experimental period, the animals were subjected to heparinization (125 UI/100 g; Akzo Organon Teknika), anesthesia with Thiopental (50 mg/Kg; Roche), and intracardiac perfusion using a 0.9% NaCl solution plus heparin (500 UI/L) and sodium nitroprusside (100mg/L; Sigma). Following perfusion, the rats were perfused with glutaraldehyde 4% (Vetec) in sodium phosphate buffer (pH 7.2 and 0.01M). After fixation, the testes were removed and weighed using a BEL Engineering balance (MARK 500/BRA) with 0.001 g precision.

The level of tissue lipid peroxidation (PLx) was assessed using the thiobarbituric acid reactive substance (TBA) test, as described by Ohkawa et al. (1979). In this method, 250 mg of hepatic and splenic tissue was homogenized using a solution of 1.51% KCl, and 0.2 ml of 1.7% sodium sulfate (SDS), 1.5 ml of 20% acetic acid solution (pH 3.5), 0.8% TBA, and 0.6 ml of distilled water were added to 0.2 ml of the tissue homogenate. The mixture was heated at 95 °C for one hour, then cooled with tap water, and finally, 5 ml of the mixture of n-butanol and pyridine (15:1) was added and centrifuged at 4000 rpm for 20 min. The organic layer was measured for absorbance at 532nm. 1,1,3,3-tetramethoxypropane (TMP) was used as an external standard, and the level of lipid peroxides (MDA) was expressed in µmol/g of protein.

### Serum testosterone analysis

Upon conclusion of the experimental period, the animals underwent heparinization (125 IU/100 g; Akzo Organon Teknika), anesthesia (Thiopental 50 mg/kg; Roche), and intracardiac perfusion with a 0.9% NaCl solution supplemented with heparin (500 IU/L) and sodium nitroprusside (100 mg/L; Sigma). Following this, all rats were perfused with a solution containing 4% glutaraldehyde (Vetec) in sodium phosphate buffer (pH 7.2 and 0.01 M) to fixate the tissues. The testes were subsequently excised and weighed using a precision balance (BEL Engineering MARK 500/BRA) with an accuracy of 0.001 g.

Blood samples were collected through puncture at the convergence of the cranial and caudal vena cava, centrifuged, and stored in Eppendorf plastic containers (two per sample) in a domestic freezer at -20°C. Hormonal levels were evaluated using the enzyme-linked immunosorbent assay (ELISA) method with absorbance reading at 405 nm, following the protocol outlined by Dias et al. (2020). Specifically, a 66.7 µL of the polyclonal anti-testosterone R156/7 antibody (Coralie Munro, University of California, Davis, USA) was diluted in 5 mL of coating buffer (Na_2_CO_3_, NaHCO_3_, ultra-pure H_2_O, pH adjusted to 9.6), and 50 µL of the antibody solution was added to each well of the NUNC Immuno TM plates (Maxisorp). Afterward, the plate was sealed with plastic and kept at 4°C for up to 12 hours. Subsequently, the standard curve was prepared by serially diluting 250 µL of the standard concentration of 600 pg/50 µL of testosterone (17-hydroxy-4-androsten-3-one, Steraloids, Sigma A6950) to a concentration of 2.3 pg/50 µL in 250 µL of ELISA assay solution (NaH_2_PO_4_, Na_2_HPO_4_, NaCl, BSA - Sigma Aldrich, A7906, ultra-pure H_2_O, pH adjusted to 7.00). The hormone conjugated with the HRP enzyme (Testosterone-horseradish peroxidase) was also diluted (33.3 µL in 5 mL of the ELISA assay solution). The substrate ELISA solution was prepared by combining 40 µL of 0.5 M H_2_O_2_, 125 µL of 40 mM ABTS (Calbiochem, ABTSTM Chromophore, Diammonium Salt), and 12.5 mL of substrate ELISA solution (citric acid, ultra-pure H_2_O, pH adjusted to 4.00). For each well containing the study or control material, 100 µL was added, and the plates were covered and incubated at room temperature under agitation (Multi-Pulse Vortexer; model 099A VB4, 50/60 Hz – Glass-Cols) until the optical density of the zero wells reached a range of 0.9 to 1. The samples were read in duplicate to minimize deviations, and the intra- and inter-assay coefficient of variation was kept below 10%. The plasma testosterone levels were expressed in nMol/L, ng/mL, and through the relationship between concentration and volume (CV%) of the hormone.

### Peripheral blood cytokine dosage

Cytokine dosage in blood supernatants collected from splenic cells was determined using the Cytometric Bead Array System (CBA) technique. The CBA was used for the quantitative measurement of the cytokines TNF-α, IL-2, IL-4, IL-6, IL-10, IL-17, IFN-γ by using BD™ Cytometric Bead Array (CBA) Mouse Th1/Th2/Th17 CBA Kit (Beckton Dickson) as recommended by the manufacturer with modifications in the final reaction volume to 60 uL (25 uL of beads mix, 25 uL of sample and 25 uL of detection reagent). The beads (2700 events) were acquired using the FACSCalibur flow cytometer (Beckton Dickson), located in the Technological Platforms Center (NPT)/IAM/Fiocruz, through the CellQuestPro software (Beckton Dickson) and analyzed in the FCAP Array 3.1 software (Beckton Dickson).

### Statistical analysis

The results were evaluated for normality using the Shapiro-Wilk test, followed by an analysis of variance (ANOVA) and the Student Newman-Keus test. STATISTICA for WINDOWS 3.11 software was used. Pearson’s correlation was used to assess the relationship between two variables. Differences were significant when P < 0.05. All results were expressed as mean and standard deviation. Data were expressed as means and standard deviation. Pearson’s correlation analysis was also performed between pairs of variables. The significance obtained in the linear correlation was evaluated, establishing that there is a high intensity correlation between the variables with low intensity with r < 0.30, medium intensity with 0.31 < r < 0.60 and high intensity with r > 0.61. A 5% probability level was used for all tests.

## Results

### Assessment of body and testicular weights

According to Table 1, we can observe that there was no significant variation between the different experimental groups in terms of body weight.

**Table 1:** Body weight (g) of control Wistar rats and rats subjected to testicular heat stress, treated and not treated with different doses of TAD, and evaluated at 7, 15 and 30 days after heat stress (Mean ±Standard deviation).

Days	Control HS (n=6)	HS + Tadalafil (0.9 mg.Kg^-1^) (n=6)	HS+ Tadalafil (1.8 mg.Kg^-1^) (n=6)	*p*
**7**	236.00 ± 32.49	225.83 ± 23.04	243.67 ± 44.56	0.675
**15**	250.83 ± 13.12	231.83 ± 18.90	242.50 ± 21.13	0.221
**30**	280.66 ± 27.20	270.66 ± 41.54	272.83 ± 45.08	0.896

### Testicular weight

Testicular weight on day 7 post-heat shock was reduced by 17 and 19.5% in the control group and treated with 0.9 mg.Kg^-1^, respectively compared to animals receiving during the same period, 1.8 mg.Kg^-1^ of TAD. In the other periods, there was no difference between the testicular weights of the different experimental groups. However, we can see a 40% reduction in testicular weight in the control group and treated with 0.9 mg.Kg^-1^, between the 7^th^ and 15^th^ days. In animals subjected to heat shock and treated with the highest dose of TAD, the reduction between days 7 and 15 was 51.7%. In the interval between days 15 and 30 of the experimental period, there was no change in testicular weight between the times studied (Table 2).

**Table 2:** Testicular weight (g) of control Wistar rats and rats submitted to testicular heat stress, treated or not with different doses of TAD, and evaluated at 7, 15 and 30 days after heat stress (Mean ± standard deviation).

Days	Control HS (n=6)	HS + Tadalafil (0.9 mg.Kg^-1^) (n=6)	HS+ Tadalafil (1.8 mg.Kg^-1^) (n=6)	*p*
**7**	0.97 ± 0.10	0.95 ± 0.09	1.18 ± 0.09	0.015
**15**	0.58 ± 0.04	0.58 ± 0.47	0.57 ± 0.09	0.952
**30**	0.58 ± 0.10	0.58 ± 0.01	0.57 ± 0.09	0.952

HS: heat stress.

### Histopathological analysis

**Figure 1:**
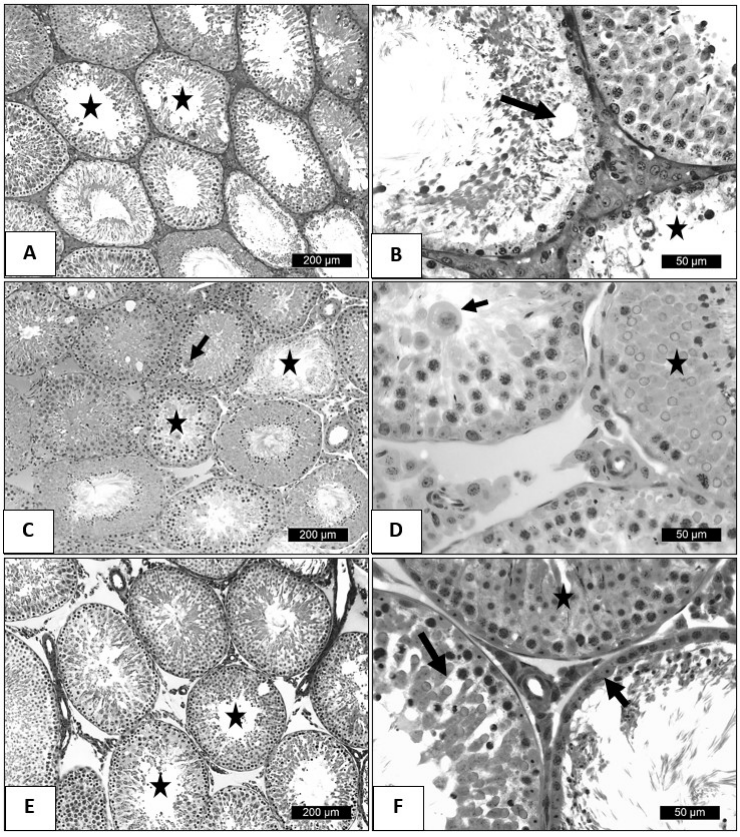
Photomicrographs of adult Wistar rat testicles subjected to testicular heat shock at 7 days of age and treated with tadalafil and control group. (A) Seminiferous tubule of the control group subjected to heat shock in a degeneration process (star); (B) Detail of seminiferous tubule of the control group subjected to heat shock. Note the vacuolization of Sertoli cells (arrow) and reduction of the germinal epithelium; (C) Seminiferous tubule of the group treated with 0.9 mg/kg subjected to heat shock, showing presence of rounded giant cells (arrow) and reduction of the seminiferous epithelium (star); (D) Detail of seminiferous tubule of the group treated with 0.9 mg/kg subjected to testicular heat shock. Note the germinal epithelium with shed cell in the lumen and tubules with normal spermatogenesis (star); (E) Seminiferous tubules of the group treated with 1.8 mg/kg of tadalafil subjected to heat shock. Note the preservation of the germinal epithelium (star); (F) Seminiferous tubules of the group treated with 1.8 mg/kg of tadalafil subjected to heat shock. Note the tubule with intact germinal epithelium (star), loosening of germinal cells (arrow), and germinal cells only in the basal compartment (short arrow).

According to histopathological findings observed in the testicular parenchyma on the 7^th^ day after the thermal stress, the animals in the control group had lesions in the seminiferous epithelium compatible with testicular degeneration. Among the main alterations of the germinal epithelium, it was possible to notice vacuolation of Sertoli cells, syncytial giant cells of rounded spermatids. Some tubules had epithelium with little vacuolization and retained most of the germ cells, while others were made up only of Sertoli cells and germ cells from the basal compartment (Figure 1A and 1B).

On the seventh day, the testis of the animals subjected to heat stress treated with 0.9 mg.Kg^-1^ (Figure 1C and D) and 1.8 mg.Kg^-1^ (Figure 1E and F) of TAD had few areas of lesions compatible with testicular degeneration. Despite the existence of changes in the germinal epithelium such as spermatocyte cell death, Sertoli cell vacuolation and desquamation of the germinal epithelium, it was possible to observe preserved seminiferous tubules throughout the testicular parenchyma. At these TAD dosages and during the evaluated period, it seems that there was an inhibition of the mechanisms that trigger degeneration by testicular thermal stress.

**Fig. 2:**
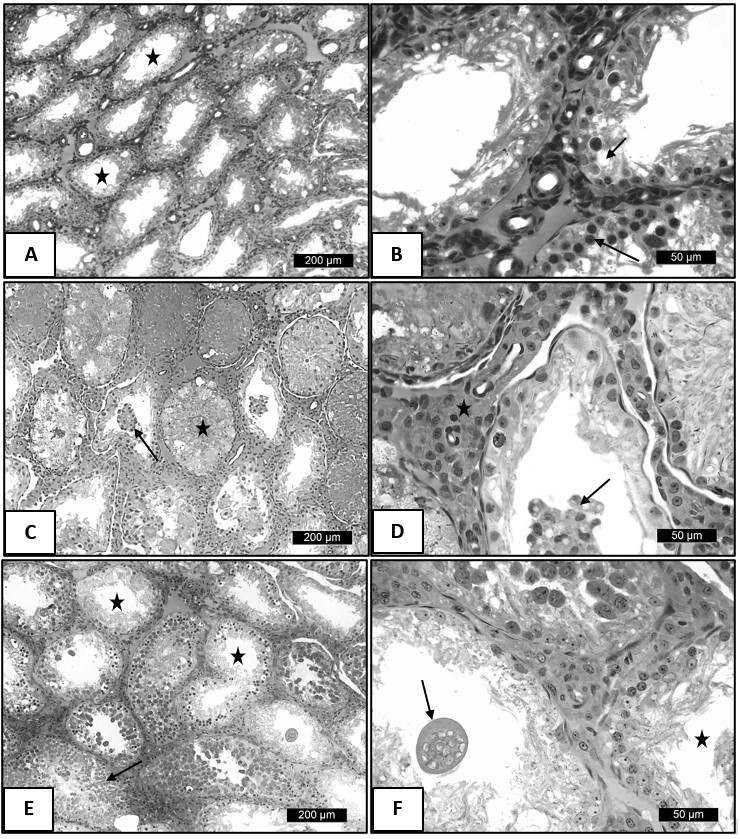
Photomicrographs of adult Wistar rat testicles subjected to testicular heat shock at 15 days, following the same procedures described earlier. (A) Note the seminiferous tubule of the control group subjected to heat shock with irregular contour and reduction of the germinal epithelium (star); (B) Detail of seminiferous tubule of the control group subjected to heat shock. Note germinal cells in the basal region of the tubule (arrow). Vacuolization of Sertoli cells (short arrow); (C) Transverse section of seminiferous tubules of the group treated with 0.9 mg/kg subjected to heat shock. Note seminiferous tubule without lumen (star) or with atrophic germinal epithelium and containing germinal cells in the lumen (arrow); (D) Detail of seminiferous tubule of the group treated with 0.9 mg/kg subjected to testicular heat shock. Note atrophy of the germinal epithelium and shed germinal cells in the lumen (arrow); Increased interstitial compartment and number of Leydig cells (star); (E) Seminiferous tubules of the group treated with 1.8 mg/kg of tadalafil subjected to heat shock. Note seminiferous tubule containing germinal cells (arrow) and others with few cells (star); (F) Transverse section of seminiferous tubules of the group treated with 1.8 mg/kg of tadalafil subjected to heat shock. Note in detail the seminiferous tubule with syncytial giant cell (arrow), and adjacent tubule with germinal epithelium containing germinal cells in the basal compartment (star).

Fifteen days after the testicular thermal stress, in the animals of the control group, it was possible to evidence seminiferous tubules of the control group submitted to thermal shock with irregular outline and reduction of the germinal epithelium. Furthermore, germ cells were detected only in the tubular basal compartment (arrow) and Sertoli cells showed intense vacuolation (Figure 2A and B). Animals subjected to heat stress and treated for 15 days with 0.9 mg.Kg^-1^ of TAD had seminiferous tubules without lumen or with atrophic germinal epithelium and containing desquamated germ cells in the lumen (Figure 2C). An increase in the interstitial compartment and number of Leydig cells was also observed (Figure 2D). The animals that received the highest dose of TAD had seminiferous tubules containing germ cells and others with few cells. In these, giant syncytial cells and germinal epithelium were observed with germinal cells only in the basal compartment (Figure 2 E and F).

**Figure 3:**
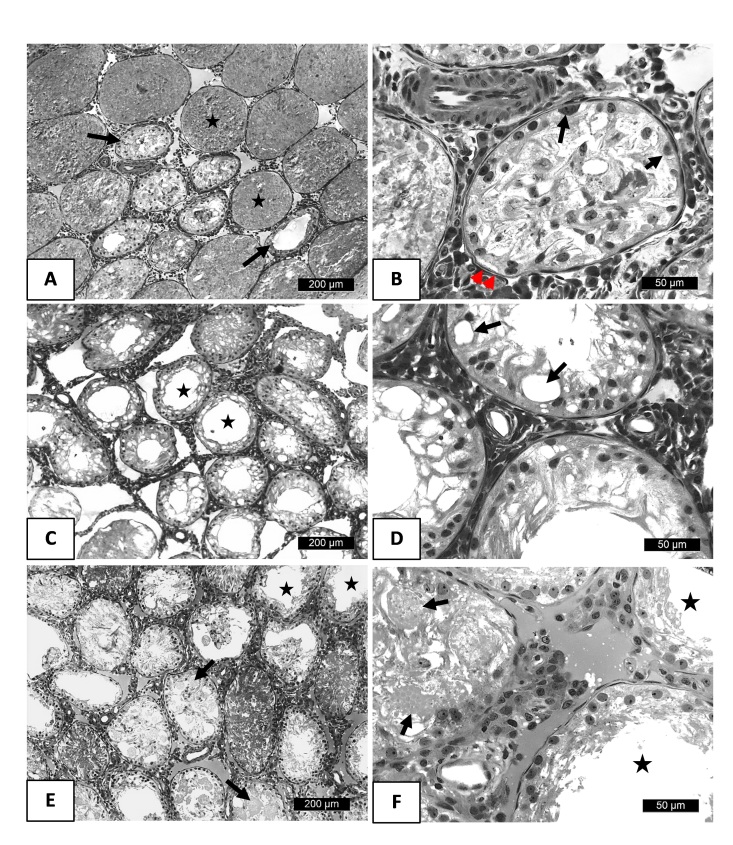
Photomicrographs of adult Wistar rat testicles subjected to testicular heat shock at 30 days of age and treated with tadalafil, compared to the control group. (A) Transverse section of seminiferous tubules of the control group. Note tubules without lumen, filled with degenerated germinal cells (star). Tubules with lumen show atrophy of the germinal epithelium (arrow); (B) Detail of seminiferous tubule of the control group. Note few basal germinal cells (arrow), Sertoli cells (short arrow), and thickening of the basal membrane (arrowhead); (C) Transverse section of seminiferous tubules of the group treated with 0.9 mg/kg subjected to heat shock. Note seminiferous tubules without germinal cells and vacuolization of Sertoli cells (star); (D) Detail of seminiferous tubules of the group treated with 0.9 mg/kg subjected to testicular heat shock. Note seminiferous epithelium without germinal cells, only Sertoli cells with cytoplasmic vacuolization (arrow); (E) Transverse section of seminiferous tubules of the group treated with 1.8 mg/kg of tadalafil subjected to heat shock. Note seminiferous tubules containing degenerated germinal cells in the tubular lumen (arrow) or without germinal cells (star); (F) In detail, observe seminiferous tubules of the group treated with 1.8 mg/kg of tadalafil subjected to heat shock. Note seminiferous tubules with syncytial cells in necrosis (arrow) and tubules containing only Sertoli cells and basal germinal cells (star).

After 30 days of testicular thermal stress in the animals of the control group, the degenerative process of the parenchyma of this organ became quite pronounced with the aggravation of the previously reported injuries. (Figure 3A and B). The seminiferous tubules were obliterated with degenerating germ cells; luminal tubules had germinal epithelial atrophy with few basal germ cells, vacuolated Sertoli cells, and basement membrane thickening.

Animals receiving 0.9 mg.Kg^-1^ of TAD during 30 days after heat shock had seminiferous tubules without germ cells and with vacuolation of Sertoli cells (Figure 3C and D). In animals treated with 1.8 mg.Kg^-1^ of DAT for 30 days, some seminiferous tubules also showed lesions compatible with the degenerative process resulting from the application of heat to the testicular tissue. The seminiferous tubules contained degenerated germ cells in the tubular lumen or were devoid of germ cells. In other tubules, rounded spermatid syncytial cells undergoing necrosis in the lumen or tubules containing only Sertoli and basal germ cells were found (Figure 3E and F).

### Serum testosterone dosage

**Figure 4:**
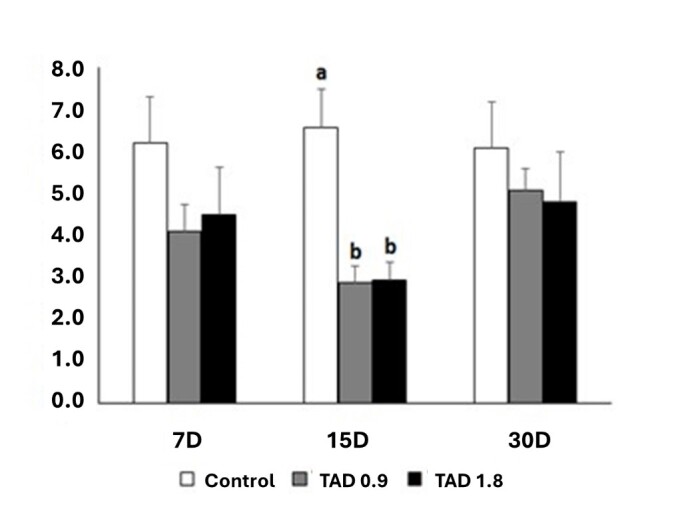
Plasma testosterone (ng/ml) of Wistar rats treated and not treated with different doses of DAT, after testicular thermal stress and evaluated at 7, 15 and 30 days post-stress (Mean ± standard error). Different letters indicate statistical difference (p<0.05).

Plasma testosterone levels did not differ on day 7 post-heat shock between the control and TAD-treated groups. However, in animals that received 0.9 mg.Kg^-1^ was found 34% reduction in this parameter in relation to the control. On the other hand, with the dosage of 1.8 mg.Kg^-1^ the reduction was 27.5% compared to the control. Despite the absence of difference or statistical trend between groups, testosterone levels were lower in this post-heat shock phase, with the administration of DAT (Figure 4; Table 3).

**Table 3:** Mean values and standard deviation of serum testosterone concentration in Wistar rats submitted to testicular thermal stress and treated or not with different doses of TAD

**Groups**	**Time**			**General mean**
	**T-7 min**	**T-15 min**	**T-30 min**	
	**Testosterone (ng/mL)**			
Controle HS	6.19 ± 1.11	6.55±0.91	6.08 ± 1.08	6.27 A
HS+Tadalafil (0.9 mg/kg^-1^)	4.08 ± 0.65	2.85±0.41	5.05 ± 0.62	3.99 B
HS+Tadalafil (1.8 mg/kg^-1^)	4.49 ± 1.11	2.91 ± 0.45	4.78 ± 1.20	4.63 B
Média Geral	4.92a	4.10a	5.31a	

Uppercase letters in the column and lowercase letters in the row differ at the 5% level of probability. “p” level of the Groups factor = 0.0088. Time factor “p” level = 0.1267. “p” level of the G x T Interaction factor = 0.0088. Level of “p” of the Times factor = 0.2321.

On day 15 post-heat shock, plasma testosterone levels had an average reduction of 56% in the TAD-treated groups compared to the control.

On the 3^rd^ moment of evaluation of the plasmatic level of testosterone (day 30), there was no difference between the experimental groups. However, we can see an average recovery in serum testosterone levels of around 42% compared to that observed on day 15 in the groups treated with TAD (Table 3). Higher serum testosterone concentrations were observed in animals in the control group compared to the other experimental groups (p=0.0088). The thermal stress model associated or not with the use of DAT at different doses did not influence the different observation times (Table 3; p=01267).

### Hepatic/splenic oxidative stress and assessment of splenic cytokines

**Figure 5:**
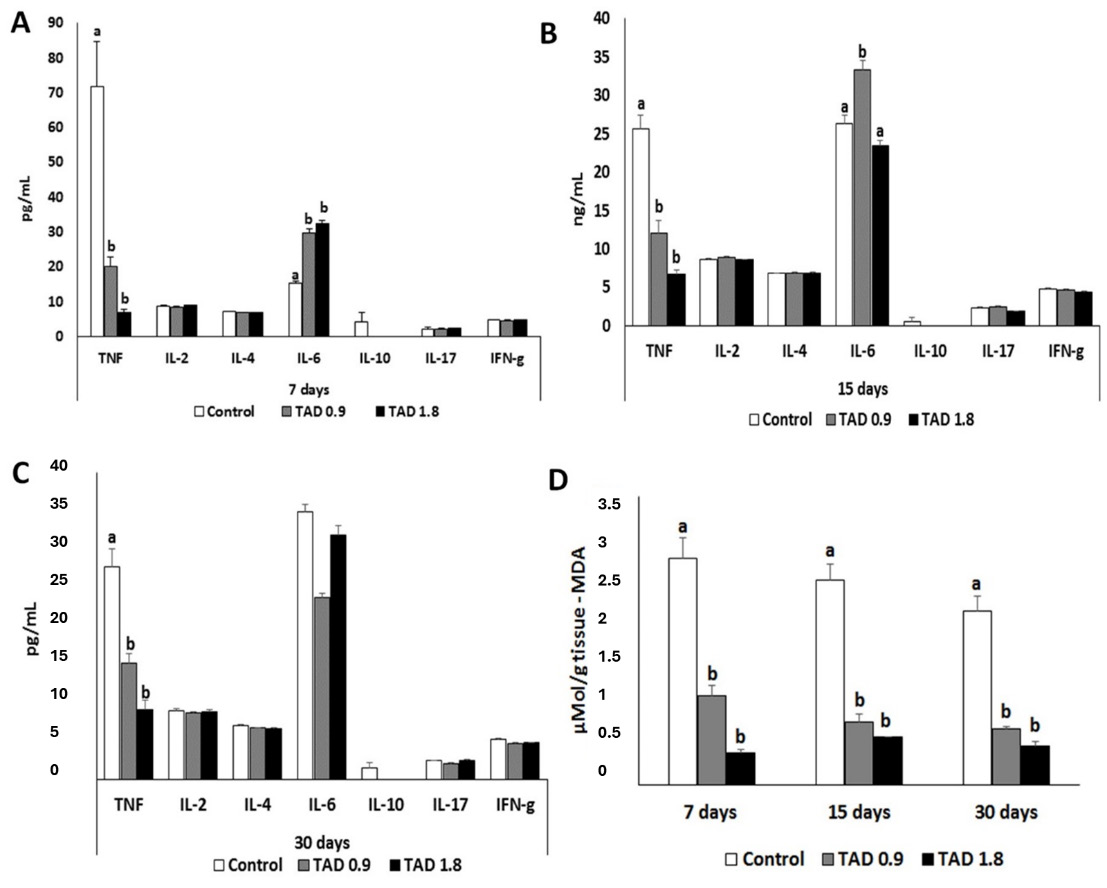
Values of splenic cytokines and determination of oxidative stress in the liver/spleen of Wistar rats submitted to testicular thermal stress, treated or not with different doses of DAT. (A) Analysis of cytokines TNF-α, IL-2, IL-4, IL-6, IL-10, IL-17, INF- γ in spleen cells from control Wistar rats and those submitted to testicular thermal stress, treated or not with different TAD doses on day 7 post-heat shock; (B) Analysis of cytokines TNF-α, IL-2, IL-4, IL-6, IL-10, IL-17, INF-? in spleen cells from control Wistar rats and those submitted to testicular thermal stress, treated or not with different TAD doses on day 15 post-heat shock; (C) Analysis of cytokines TNF-α, IL-2, IL-4, IL-6, IL-10, IL-17, INF- γ in spleen cells from control Wistar rats and those submitted to testicular thermal stress, treated or not with different TAD doses on day 30 post-heat shock; (D) Mean values of nitric oxide (mMol/mL) in the liver tissue of Wistar rats subjected to testicular heat stress treated or not with different doses of TAD on days 7, 15 and 30, through reaction to thiobarbituric acid (TBARs). Different letters shows that there was a significant difference in the comparison, p<0.05).

Table 4 and Figure 5 shows the nitric oxide values in the hepatic/splenic tissue homogenate of control Wistar rats and rats submitted to testicular thermal stress and treated with different doses of TAD, as well as the levels of cytokines: TNF-α, IL -2, IL-4, IL-6, IL-10, IL-17, INF-α obtained from splenic cell supernatants. In agreement with the results observed in figures 2A-C, the levels of TNF-α in the groups treated with 0.9 and 1.8 mg. Kg-1 of TAD reduced at all times studied in relation to animals submitted to testicular heat stress without treatment. IL-6 levels on day 7 post-heat shock increased in animals treated with 0.9 and 1.8 mg.Kg^-1^ of TAD in relation to the control group. At the second time point of post-thermal shock evaluation, interleukin-6 increased only in the group treated with 0.9 mg.Kg^-1^. On day 30 post-heat shock, no change in IL-6 levels was observed between the experimental groups (Figure 5A-C). Lipid peroxidation levels are depicted in Figure 4D. According to the results, a reduction in lipid peroxidation levels was observed at all times evaluated after thermal shock, in the experimental groups that received TAD.

**Table 4:** Mean values, standard deviation and significance level (Pr>F) of interleukins and nitric oxide in Wistar rats submitted to testicular heat stress and treated with different doses of TAD (0.9 e 1.8 mg.Kg^-1^).

**Variables**	**Groups**			**Pr>F**
	**Control HS**	**HS+Tadalafil** **(0.9 mg.Kg^-1^)**	**HS+Tadalafil** **(1.8 mg.Kg^-1^)**	
TNF-α (pg/mL)	42.04 ± 5.61 a	15.81 ± 1.88 b	7.63 ± 0.86 c	<.0001
IL-2 (pg/mL)	8.78 ± 0.15	8.71 ± 0.28	8.80 ± 0.15	0.7228
IL-4 (pg/mL)	6.97 ± 0.11	6.81 ± 0.16	6.78 ± 0.09	0.0521
IL-6 (pg/mL)	22.88 ± 0.87 b	30.00 ± 0.96 a	30.96 ± 0.95 a	<.0001
IL-10 (pg/mL)	1.91 ± 0.98 a	0.00 ± 0.00b	0.00 ± 0.00 b	0.0085
IL-17 (pg/mL)	2.22 ± 0.28	2.22 ± 0.11	2.26 ± 0.07	0.8944
INF-γ (Unidade)	4.91 ± 0.11 a	4.66 ± 0.09 b	4.62 ± 0.13 b	0.0062
Nitric oxide (mMol/mL)	2.45 ± 0.21 a	0.84 ± 0.08 b	0.48 ± 0.04 c	<.0001

Different letters shows that there was a significant difference in the comparison, p<0.05.

The values of TNF-α and Nitric Oxide were higher in the animals in the Control HS group, followed by lower means in the animals in the HS+TAD (0.9 mg.Kg^-1^) and HS+TAD (1.8 mg.Kg^-1^) groups, respectively. As for IL-10 and INF-α values, the highest values were also observed in the animals of the HS control group, however differing from the other groups. The opposite behavior occurred with the serum concentration of IL-6, in which lower averages were observed in animals in the control group, when compared to the other groups (HS+TAD 0.9 mg.Kg^-1^ and HS+TAD 1.8 mg.Kg^-1^). The interleukins IL-2, IL-4 and IL-17 were not influenced by the treatments used in the experiment (Table 4).

In the interaction analysis between groups and moments, interaction was recorded for the variables: TNF-α (p<.0001), IL-6 (p<.0001), IL-17 (p<.0001) and Nitric oxide (p=0.0359). In the absence of interaction Groups x Times, significant variations were registered, in the group factor, for the following INF-α variables (p=0.0062). Regarding the time factor, significant variation was observed for INF-α (p=0.0190). Interleukins IL-2 and IL-4 were not influenced by the effects of groups, times or group x time interaction (p>0.05) (Table 5).

**Table 5:** Significance levels of the analysis of variance for the variation factors (Groups and Times) and their interaction (Groups x Times) of the set of variables of Wistar rats submitted to testicular thermal stress and treated or not with different TAD doses.

**Variables**	**Unit**	**Pr>F**	**ANOVA Variation Factors**		
			**Groups**	**Time**	**G x T**
TNF-α	(pg/mL)	<.0001	<.0001	<.0001	<.0001
IL2	(pg/mL)	0.3343	0.7228	0.4641	0.1458
IL4	(pg/mL)	0.1176	0.0521	0.3988	0.2456
IL6	(pg/mL)	<.0001	<.0001	<.0001	<.0001
IL10	(pg/mL)	0.0424	0.0085	0.4678	0.5487
IL17	(pg/mL)	0.0008	0.8944	0.2545	<.0001
INF-γ	(pg/mL)	0.0031	0.0062	0.0190	0.1091
Nitric oxide	(mMol/mL)	<.0001	<.0001	0.0469	0.0359

According to Table 6, the interleukins IL-2, IL-4 and IL-10 were not influenced by the treatments used in the experiment, but the INF-α had a time effect, with lower averages being observed in the animals at times T7 and T -15 when compared to the highest T-30 time average. As for the interleukins TNF-α, IL-6, IL-10 and Nitric Oxide, these showed interaction effect Groups x Times.

**Table 6:** Mean values, standard deviation and variation factors Time and Interaction Groups x Times of serum concentration of testosterone, nitric oxide and interleukins in Wistar rats submitted to testicular thermal stress and treated or not with different doses of TAD.

**Groups**	**Time**			**Variation Factors**	
	**T-7 day**	**T-15 day**	**T-30 day**	**Time**	**G x T**
	**TNF-α (pg/mL)**				
Control HS	71.83±12.81aA	25.68±1.75bA	28.61±2.32bA		
HS+Tadalafil (0.9 mg/kg^-1^)	20.21±2.67aB	12.10±1.63bB	15.12±1.34bB	<.0001	<.0001
HS+Tadalafil (1.8 mg/kg^-1^)	7.00±0.89aB	6.76±0.53aC	9.13±1.16aC		
Mean general	33.01	14.85	17.62		
	**IL-2 (pg/mL)**				
Control HS	8.72±0.15	8.68±0.09	8.95±0.20		
HS+Tadalafil (0.9 mg/kg^-1^)	8.51±0.16	8.89±0.09	8.72±0.06	0.4641	0.1458
HS+Tadalafil (1.8 mg/kg^-1^)	8.94±0.12	8.58±0.10	8.8±0.247		
Mean general	8.72	8.71	8.85		
	**IL-4 (pg/mL)**				
Control HS	7.06±0.11	6.82±0.06	7.03±0.16		
HS+Tadalafil (0.9 mg/kg^-1^)	6.83±0.06	6.90±0.10	6.70±0.09	0.3988	0.2456
HS+Tadalafil (1.8 mg/kg^-1^)	6.83±0.11	6.84±0.07	6.66±0.10		
Mean general	6.91	6.85	6.80		
	**IL-6 (pg/mL)**				
Control HS	15.30±0.47cC	22.75±1.10bC	30.58±1.03aA		
HS+Tadalafil (0.9 mg/kg^-1^)	29.85±1.07bB	33.28±1.31aB	26.88±0.50bB	<.0001	<.0001
HS+Tadalafil (1.8 mg/kg^-1^)	32.55±0.87aA	28.53±0.66bA	31.81±1.33aA		
Mean general	25.90	28.19	29.76		
	**IL-10 (pg/mL)**				
Control HS	4.18±1.66	0.55±0.55	1.00±0.72		
HS+Tadalafil (0.9 mg/kg^-1^)	0.00±0.00	0.00±0.00	0.00±0.00	0.4678	0.5487
HS+Tadalafil (1.8 mg/kg^-1^)	0.00±0.00	0.00±0.00	0.00±0.00		
Mean general	1.39	0.18	0.34		
	**IL-17 (pg/mL)**				
Control HS	1.20±0.70bA	2.31±0.10aA	2.36±0.05aA		
HS+Tadalafil (0.9 mg/kg^-1^)	2.18±0.16aA	2.40±0.12aA	2.08±0.06aB	0.2545	<.0001
HS+Tadalafil (1.8 mg/kg^-1^)	2.37±0.08aA	1.91±0.02bB	2.48±0.12aA		
Mean general	2.18	2.21	2.31		
	**INF-γ (pg/mL)**				
Control HS	4.70±0.14	4.81±0.09	5.22±0.11		
HS+Tadalafil (0.9 mg/kg^-1^)	4.61±0.11	6.81±0.08	4.69±0.07	0.0190	0.1091
HS+Tadalafil (1.8 mg/kg^-1^)	4.69±0.19	4.41±0.10	4.75±0.09		
Mean general	4.67b	4.63b	4.89a		
	**Nitric oxide (mMol/mL)**				
Control HS	2.75±0.25aA	2.48±0.20aA	2.11±0.18aA		
HS+Tadalafil (0.9 mg/kg^-1^)	1.08±0.12aB	0.76±0.10bB	0.68±0.02bB	0.0469	0.0359
HS+Tadalafil (1.8 mg/kg^-1^)	0.38±0.05aC	0.58±0.00aB	0.47±0.06aB		
Mean general	1.40	1.27	1.09		

HS: heat stress; TAD: tadalafil. Uppercase letters in the column and lowercase letters in the row differ at the 5% level of probability.

The highest means of TNF-α were observed at time T-7 of testicular heat stress for animals in the Control HS and HS+TAD (0.9 mg.Kg^-1^) groups, and these differed from the other times (T-15 and T -30). As for the means of the HS+TAD group (1.8 mg.Kg^-1^), at different times, these were similar at the three observation times. Regarding the analysis of the means of the groups within each time, it was found that higher means of TNF-α were recorded in animals at times T-7, T-15 and T-30 for animals in the HS Control group and lower means for the other treatments (HS+TAD 0.9 mg.Kg^-1^ and HS+TAD 1.8 mg.Kg^-1^) (Table 6).

IL-6 gradually increased for animals in the HS control group over time, while for animals in the HS+TAD group (0.9 mg.Kg^-1^) the highest average was recorded at time T-15 in relation to the others, the which occurred on the contrary in the animals of the HS+TAD group (1.8 mg.Kg^-1^). With regard to the analysis of the means of the groups within each time, it was found that there was a chronological increase in IL-6 in the groups at times T-7 and T15, but at T-30 and only in the HS+TAD group (0.9 mg.Kg^-1^), the lowest mean was recorded compared to the other groups (Table 6).

IL-17 had a lower concentration in animals in the HS control group at time T-7 and higher at other times; as for the HS+TAD animals (0.9 mg.Kg^-1^), there was no significant variation in the other times, however with the HS+TAD animals (1.8 mg.Kg^-1^), there was a reduction in the T-15 time when compared to with the T-7. Regarding the analysis of the means of the groups within each time, it was observed that the lowest means of IL-17 was recorded in T-15 for animals in the HS+TAD group (1.8 mg.Kg^-1^) and in the time T- 30 the lowest average was registered in the HS+TAD group (0.9 mg. Kg^-1^) (Table 6).

Nitric oxide had its highest concentration in animals from the HS+DAT group (0.9 mg/kg) at time T-7 compared to the others, while a decrease was observed at the other times (T-15 and T-30). Regarding the analysis of the averages of the groups within each time, the highest averages were observed in the animals of the HS control group, in the three observation times, and that these decreased in the other treatments, in the respective times (Table 6).

**Figure 6:**
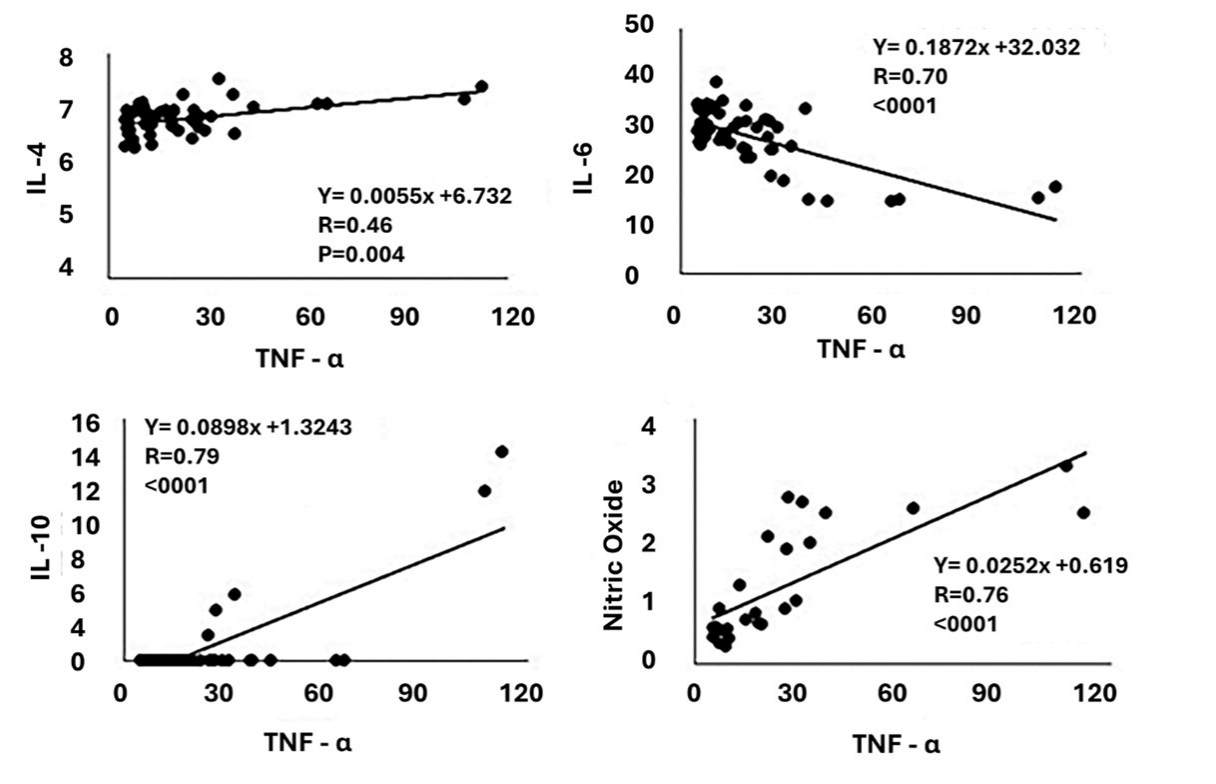
Graphical representation of Pearson’s correlation and respective levels of significance of serum NTF concentrations with IL-4, IL-6, IL-10 and Nitric Oxide in Wistar rats subjected to testicular thermal stress and treated with different doses of TAD.

**Figure 7:**
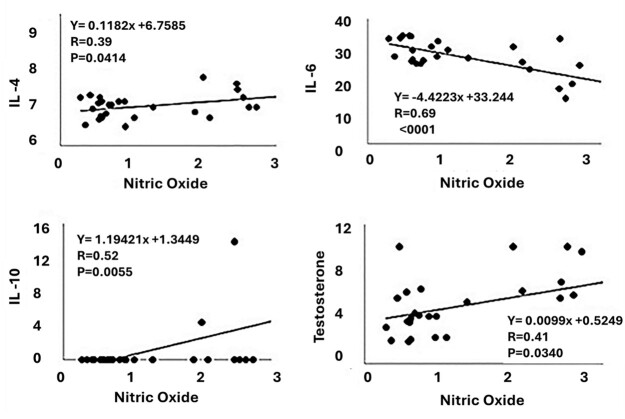
Graphical representation of Pearson’s correlation and respective levels of significance of serum concentrations of Nitric Oxide with IL-4, IL-6, IL-10 and Testosterone in Wistar rats subjected to testicular thermal stress and treated with different doses of TAD.

In the correlation analysis (Figures 6 and 7), there was a high positive correlation between TNF-α and IL-10 (r=0.79; <.0001) and Nitric Oxide (r=0.76; <.0001), but moderately positive with IL-4 (r=0.79; p=0.0004). High negative correlation was observed between TNF-α and IL-6 (r= -0.70; <.0001). High negative correlation was observed between nitric oxide and IL-6 (r= -0.69; <.0001), but moderately positive with IL-4 (r=0.39; p=0.0414), IL-10 (r=0.52; p=0.0055) and testosterone (r=0.41; p=0.0340). Finally, a moderate positive correlation was observed between IL-17 with IL-2 (r=0.33; p=0.0158) and with IL-6 (r=0.32; p=0.0164). Likewise, a moderate positive correlation was observed between IL-10 and IL-4 (r=0.42; p=0.0017), but a moderately negative correlation with IL-6 (r=-0.38; p= 0.0042).

## Discussion

Spermatogenesis is a complex phenomenon with different stages of the spermatogenic cycle, i.e. germinal, stem cell proliferation, meiotic division, and spermiogenesis, it is relatively more vulnerable to various stresses including heat stress (Lue et al., 1999; Rockett et al., 2001; Queiroz et al., 2013). Elevation of testicular temperature results in damage to spermatogenesis, the progression of this type of cell death after the elevation of temperature causes several testicular structural changes that culminate in a reduction in testicular weight (Kastelic et al., 1997, 2019; Kumagai et al., 2002; Silva et al., 2006; Queiroz et al., 2013). This parameter has a high and positive correction with the population of Sertoli cells, germ cells, the height of the germinal epithelium, the total length of seminiferous tubules, and daily sperm production (França et al., 2005).

In the present study, the highest dose of TAD for 7 days after heat shock had a positive effect on testicular weight. In the other periods, there was no difference in testicular weights between groups. On the other hand, evaluating the changes in intra-group weight between the periods studied, a 40% reduction in testicular weight was observed in the control group and treated with 0.9 mg.Kg^-1^, between the 7^th^ and 15^th^ days. In the same period, for animals subjected to heat shock that received the highest dose of TAD the reduction was 51.7%. In the interval between days 15 and 30 of the experimental period, there was no change in testicular weight between the times studied.

Degeneration of the seminiferous epithelium can occur quickly, but if the period of hyperthermia is not too long, visible regeneration can occur 60 days after exposure to heat. On the other hand, in very severe lesions where spermatogonia A is affected, azoospermia can occur (Queiroz et al., 2013).

The testicular alterations detected in the present study are consistent with those observed by Kanter (Kanter et al., 2013; Rebez et al., 2023). According to these authors, rats exposed to thermal shock had intratubular vacuoles, giant syncytial cells, pyknotic germ cells, and cells with apoptotic fragments. However, the protocol used to induce testicular changes via testicular thermal shock was a single exposure to 43 °C for 15 minutes.

Tadalafil (TAD) is a phosphodiesterase-5 (PDE5i) inhibitor commonly used in the treatment of erectile dysfunction in the current practice of urology (van Driel, 2006; Gul et al., 2020). Inhibition of phosphodiesterase (PDE) enzymes increases cGMP levels in tissue, therefore resulting in smooth muscle relaxation. There are several studies in the literature that investigate I/R injury in testicular torsion models in rats using different PDE5 inhibitors such as sildenafil and vardenafil (van Driel, 2006; Erol et al., 2009; Samidurai et al., 2023).

The use of DAT for 7 days after the heat shock, despite the existence of alterations compatible with the process of testicular degeneration, provided the preservation of seminiferous tubules throughout the testicular parenchyma. Heat stress promotes an increase in reactive oxygen species (ROS) in different organs, including the testicles (Zhang et al., 2005; Boni, 2019). Thus, the action of this specific PDE-5 inhibitor seems to have influenced the modulation of mechanisms that trigger degeneration by heat stress testis in this period, which may be directly related to the reduction of oxidative stress and anti-inflammatory properties (Ameli et al., 2018; Laxmi et al., 2019).

In the present study, animals subjected only to heat shock did not have changes in serum testosterone levels throughout the experimental period. However, according to Li et al (2009), heat stress influences serum concentrations of testicular testosterone and enzymes required for testosterone biosynthesis in Leydig cells, cytochrome P450 family 17 (CYP17), and steroidogenic acute regulatory protein ^.^(Li et al., 2015).The thermal stress protocol, by the mentioned authors, used the same temperature and twice the time used in the present study, which could cause greater damage to the Leydig cells.

There was a reduction in serum testosterone levels at T15 post-thermal stress and recovery in levels of this hormone at T30 in animals treated with TAD. Despite the antioxidant capacity of TAD, it was not able to maintain testosterone levels in the treated groups, on days 7 and 15 of the evaluation. Queiroz et al1, using the same protocol for thermal stress and different doses of pentoxifylline, a nonspecific phosphodiesterase inhibitor with anti-inflammatory properties, reducing superoxide anions and TNF-α inhibitor, did not observe a positive influence on testosterone levels in rats subjected to stress temperature, in the evaluated periods. On the other hand, in the experimental model of cyclophosphamide-induced testicular degeneration, prior administration of DAT prevented testicular dysfunction and reduction in testosterone levels in a dose-dependent manner (Yigitaslan et al., 2014). Exposure of mice to cyclophosphamide caused a reduction in 17β-HSD activity and serum testosterone levels (Pavin et al., 2018). On the other hand, heat stress induced by testicular insulation in sheep does not reduce 17β-HSD activity or testosterone levels (Escobar et al., 2019). Therefore, it is very likely that DAT directly or indirectly interfered with steroidogenesis in animals subjected to heat stress.

TNFα is a multifunctional cytokine with effects not only on the pro-inflammatory response (Fiers, 1991) but also on immunoregulatory responses (Beutler, 1995) and apoptosis (Baker and Reddy, 1998; Tiegs and Horst, 2022). In this study, TNF-α levels were reduced in all evaluation periods in animals receiving TAD. Sildenafil and tadalafil decreased pro-inflammatory cytokines (IL-4 and TNF-α), oxidative and nitrosative stress in an animal model of bronchial asthma (Laxmi et al., 2019). In the renal ischemia and reperfusion (I/R) model, sildenafil had a renoprotective effect due to the activation of antioxidant genes (Nrf2, HO-1, and NQO-1), anti-apoptotic gene (Bcl2) and attenuation of pro-inflammatory cytokines (TNF-α, IL-1β, and ICAM-1) (Zahran et al., 2015). Due to the consistent reduction of TNF-α over time in the present study, we can consider that TAD, at doses of 0.9 and 1.8 mg. Kg-1 had anti-inflammatory action, however, it was not able to inhibit the testicular degenerative process.

IL-6 is a pro-inflammatory cytokine that activates immune responses, and inflammation (Khan et al., 1992), increases the level of lipid peroxidation of the sperm membrane (French et al., 2008), and the production of nitric oxide (Lampiao and du Plessis, 2008). In thermal stress induced by varicocele, an increase in IL-6 levels over time was observed, as well as progressive testicular damage (Habibi et al., 2015).

In the present experimental model, IL-6 levels on day 7 post-heat shock increased in animals treated with 0.9 and 1.8 mg. Kg-1 of TAD with the control group. At the second time point of post-thermal shock evaluation, interleukin-6 increased only in the group treated with 0.9 mg. Kg^-1^. On day 30 post-heat shock there was no change in IL-6 levels between the experimental groups. However, in the present study, although there was no significant variation in testosterone and IL-6 (p=0.2238), a negative relationship was observed between serum IL-6 concentration and serum testosterone concentration (r= -0.17; p=0.2238).

According to Yigitaslan et al. (2014), previous treatment with increasing doses of DAT, in a model of toxic testicular degeneration by cyclophosphamide, did not influence IL-6 levels.

Renal ischemia and reperfusion (I/R) injury is a major cause of acute renal failure, which can be accompanied by inflammation and secondary tissue injury. In this model, the previous use of DAT reduced the circulating levels of the pro-inflammatory cytokines TNF-α, IL-1β, and IL-6 (Medeiros et al., 2017). Vignozzi et al. (2013) demonstrated that tadalafil has antioxidant and anti-inflammatory action, in addition to its vasodilator property, and that PDE5 inhibitors suppressed other genes induced by TNFα related to inflammation (IL-6, MCP1, IL-12, COX2).

Exogenous elevation in IL-6 levels promotes prolonged suppression of testosterone levels by inducing persistent testicular resistance to LH action or suppression of Leydig cell steroidogenesis, or both, with potentially adverse effects on male reproductive function (Tsigos et al., 1999; Acharyya, 2021). The administration of DAT after the induction of testicular injury by thermal shock did not influence the reduction of IL-6 as observed in studies whose application protocol had a more preventive purpose than blocker/tissue restorer in the testicle. Thus, it is very likely that IL-6 levels have negatively influenced plasma testosterone in this study.

In the model of acute environmental heat stress, reactive oxygen species damage the DNA of spermatocytes I and rounded spermatids, activate caspases, and alter structures in the testicular parenchyma (Houston et al., 2018). High levels of ROS (superoxide, hydroxyl, hydrogen peroxide, nitric oxide, and peroxynitrite) adversely affect normal sperm production and quality (motility, viability, and function) by interacting with membrane lipids, proteins, nuclear and mitochondrial DNA (Aprioku, 2013)

Lipid peroxidation products have been widely used as indicators of ROS generation in different I/R ischemia/reperfusion models (Granger and Kvietys, 2015). In the testicular heat stress model, lipid peroxidation levels were reduced at all times at different TAD doses. Our findings corroborate those of Ameli et al (Ameli et al., 2018), due to the increase in levels of SOD, and GPx and reduction in MDA levels, characterizing the antioxidant role of TAD. On the other hand, this reduction in lipid peroxidation did not interfere with the worsening of testicular degeneration after testicular thermal stress. Tadalafil reduces pro-oxidant activity and increases antioxidant activity (Iordache et al., 2020).

## Conclusion

Tadalafil exerted an anti-inflammatory and antioxidant effect, delaying the initial testicular degenerative process. However, it was not able to prevent the worsening of testicular lesions in subsequent periods, even with the systematic reduction in TNF-α and NO levels. This selective PDE-5 inhibitor, at the dosages employed, did not have a positive effect on the maintenance of testosterone levels in the post-thermal stress period, which would make it difficult to resume the spermatogenic process.
